# Tolerance Induction Strategies in Organ Transplantation: Current Status and Future Perspectives

**DOI:** 10.3389/ti.2025.14958

**Published:** 2025-10-07

**Authors:** Tifanie Blein, Nicolas Ayas, Soëli Charbonnier, Artur Gil, Juliette Leon, Julien Zuber

**Affiliations:** ^1^ Institut Necker Enfant Malades, Institut national de la santé et de la recherche médicale (INSERM) Unité U1151, Paris, France; ^2^ Université Paris Cité, Paris, France; ^3^ Service des Maladies du Rein et du Métabolisme, Transplantation et Immunologie Clinique, Hôpital Necker, Assistance Publique Hôpitaux de Paris (APHP), Paris, France

**Keywords:** transplantation, thymus, hematopoietic chimerism, immune tolerance induction, regulatory cell therapy, genetic engineering

## Abstract

Achieving donor-specific immune tolerance has the potential to eliminate the need for lifelong immunosuppression in transplant recipients, but translating this goal into clinical practice remains challenging. Unlike laboratory rodents, humans are exposed to a variety of pathogens that generate memory T cells, which can interfere with tolerance induction. Establishing full donor hematopoietic chimerism, whether spontaneous or induced, can support robust immune tolerance. However, it often relies on graft-versus-host (GvH) reactivity, which carries significant risks, including graft-versus-host disease (GVHD) and infection. Although non-myeloablative conditioning protocols have shown promise, their broader use is limited by concerns about toxicity and the need to carefully balance GvH responses. Mixed and transient chimerism represents a less toxic alternative, but its effectiveness in humans is hindered by limited durability and resistance from memory T cells. Thymus transplantation offers another strategy by promoting central tolerance through donor-specific thymic education of developing T cells. Regulatory cell therapies combined with reduced immunosuppression have emerged as a safer approach. Early clinical trials have yielded encouraging results. Innovations in IL-2 pathway modulation and genetic engineering, including CAR-redirected regulatory T cells, may further enhance the precision, durability, and safety of strategies aimed at achieving transplantation tolerance.

## Immune Tolerance

The concept of immune tolerance in transplantation refers to the immune system’s inability to mount an effector response selectively against donor antigens. In experimental models, the acceptance of a second graft from the same donor in the absence of immunosuppressive therapy, contrasted with the rejection of a third-party allograft, defines donor-specific tolerance. In humans, such experimental validation is not feasible. Instead, the term operational tolerance is used to describe a state in which the graft maintains normal function and histology in the absence of immunosuppressive treatment, and the recipient shows no increased susceptibility to infections, indicating overall preservation of immune competence.

Achieving a stable and robust state of donor-specific tolerance in clinical transplantation would allow for the elimination of long-term immunosuppression and its many associated complications, notably infections and malignancies. The aim of this review is not to provide an exhaustive account of all ongoing protocols, an effort already comprehensively undertaken in the report from the 6th International Sam Strober Workshop on Transplantation Tolerance [1], but rather to offer a critical and balanced perspective on current results and potential avenues for improvement.

## Challenges in Translating Tolerogenic Protocols to Clinical Transplantation

Numerous immunomodulatory strategies have successfully induced tolerance to allogeneic tissues or organs in experimental models, particularly in mice [2]. However, the translation of these tolerogenic protocols to the clinical setting has often yielded disappointing or even negative results, thus limiting the translational relevance of rodent-based models [2]. It is essential to examine the reasons behind these failures in order to adapt these strategies to the specificities of the human host.

One major difference lies in the microbial exposure of humans (and other large mammals), which contrasts sharply with the controlled environments in which laboratory rodents are bred, typically under specific pathogen-free (SPF) or even more stringent conditions (SOPF) [3]. As a consequence, humans develop a substantial compartment of memory T cells [3], including donor-reactive memory T cells generated through heterologous immunity [4]. These cells contribute to a relative resistance to the induction of transplantation tolerance [5]. It is well established that laboratory mice exhibit a naïve-to-memory T cell ratio comparable to that of human neonates [3]. Remarkably, mice derived from pet stores or farms, by contrast, show a distribution of memory T cells within lymphoid organs and peripheral tissues similar to that observed in adult humans [3]. Furthermore, infection of a laboratory mouse with a single pathogenic virus can render it refractory to tolerance induction via peripheral immunomodulation, an approach otherwise highly effective in uninfected animals [6]. A dose-dependent effect has also been demonstrated: co-infection with multiple pathogens further increases resistance to tolerance induction [6].

In addition, despite numerous promising studies [7], there are still no universally validated biomarkers of tolerance in transplantation. This lack of reliable markers continues to preclude the safe and personalized tapering or withdrawal of immunosuppressive therapy [1, 8, 9].

## Spontaneous Mixed Hematopoietic Chimerism Following Solid Organ Transplantation

In 2008, a landmark publication reported the spontaneous development of full hematopoietic chimerism in a 9-year-old girl following liver transplantation, in the absence of any myeloablative conditioning regimen [10]. This case demonstrated, first, that a transplanted liver can harbor a sufficient number of hematopoietic stem cells (HSCs) to support complete, multilineage, and durable hematopoiesis [10]. More importantly, it highlighted the capacity of graft-versus-host (GvH) reactivity, mediated by donor-derived T cells, to mimic the effects of bone marrow transplant conditioning.

This facilitating role of GvH reactivity includes two key mechanisms [1]: suppression of the host-versus-graft (HvG) immune response, and [2] clearance of hematopoietic niches via destruction of host HSCs, thereby enabling donor cell engraftment [10].

We recently reported a similar case following isolated kidney transplantation [11, 12]. Durable engraftment of HSCs derived from the renal graft was established in the recipient’s bone marrow [12]. In this case as well, the induction of full chimerism was associated with robust GvH reactivity [12]. In both instances, immunosuppressive therapy was successfully discontinued without subsequent graft rejection, despite restoration of immune competence, thereby meeting the criteria for operational tolerance [10, 12].

To further elucidate the mechanisms linking GvH reactivity and hematopoietic chimerism, the group led by Megan Sykes at Columbia University investigated patients undergoing intestinal and multivisceral transplantation, in whom graft survival without rejection has been shown to correlate with the volume of transplanted tissue [13, 14]. A direct relationship was identified between the number of transplanted organs and the extent of hematopoietic chimerism observed post-transplant [15]. Notably, donor-derived T lymphocytes from visceral grafts were found to mediate GvH reactivity that supported the persistence of hematopoietic chimerism not only in the graft itself [16], but also in the recipient’s peripheral blood and bone marrow [17, 18].

Collectively, these observations in solid organ transplant recipients, none of whom underwent myeloablative conditioning, underscore the critical role of GvH reactivity in the establishment and maintenance of hematopoietic chimerism.

## Induction of Full Donor Hematopoietic Chimerism

**FIGURE 1:**
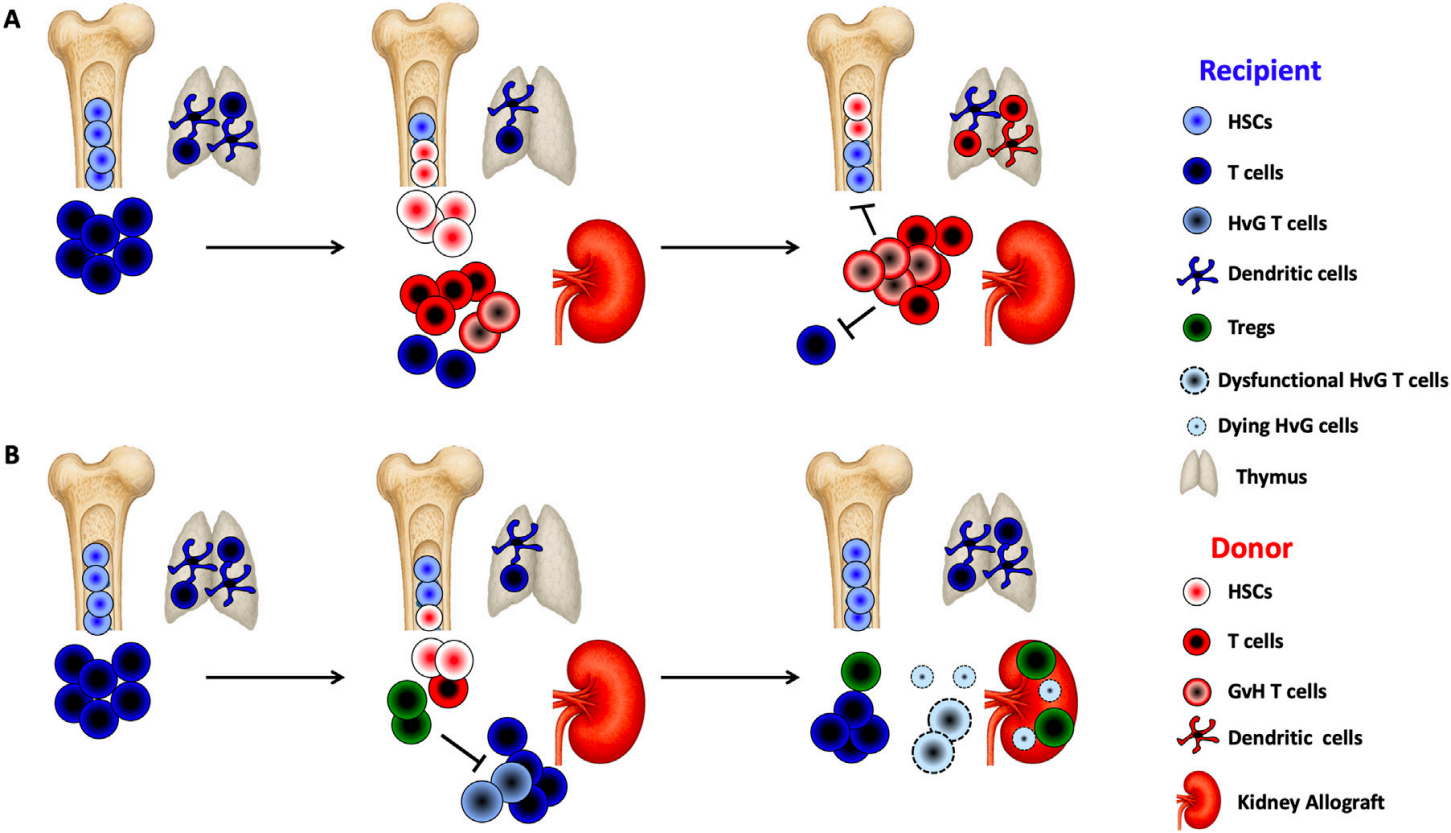
Chimerism-based transplant tolerance **(A)** Sustained Full Chimerism: Following non-myeloablative conditioning, recipients receive a large number of donor CD34^+^ hematopoietic stem cells (HSCs) and donor T cells along with the kidney allograft. Graft-versus-host (GvH) reactivity may promote the expansion of donor T cells, which eliminate recipient T cells and hematopoietic cells, thereby creating space in the bone marrow for donor HSCs to engraft. Once engrafted, donor HSCs continuously supply the thymus with T cell precursors and dendritic cell progenitors, promoting central donor-specific tolerance. These conditions support the establishment of sustained full chimerism. **(B)** Transient Mixed Chimerism: After non-myeloablative conditioning with T cell-depleting induction, recipients receive unfractionated donor bone marrow, including HSCs and T cells, alongside the kidney allograft. The conditioning regimen induces lymphopenia while sparing regulatory T cells (Tregs), leading to early Treg expansion. In the presence of donor antigen, this Treg-dominant environment suppresses the activation of host-versus-graft (HvG)-reactive T cells and fosters peripheral deletion of donor-reactive T cells. These mechanisms enable the development of transient mixed chimerism.

The induction of stable immune tolerance associated with full donor chimerism was first achieved through sequential transplantation of hematopoietic progenitor cells and a kidney from the same HLA-incompatible donor in patients undergoing treatment for hematologic malignancies [19]. When full donor chimerism is established, donor-derived dendritic cells colonize the recipient’s thymus (Figure 1). This allows newly developing thymocytes to undergo negative selection if they are reactive to donor or recipient antigens, presented respectively by donor dendritic cells and recipient medullary thymic epithelium (Figure 1). Tolerance is thus predominantly mediated through central mechanisms and requires a stable, long-term dominance or completeness of donor hematopoiesis [20].

However, this approach typically necessitates myeloablative conditioning, which carries unacceptable toxicity in patients without malignancy. In transplantation, several strategies have been developed to induce full chimerism, and thereby stable tolerance, without resorting to myeloablation [20].

The first such protocol, developed at Stanford University, combines total lymphoid irradiation, anti-thymocyte globulin (ATG), and infusion of a limited number of donor T cells (1 × 10^6^/kg). This approach achieved durable chimerism and successful immunosuppression withdrawal in more than 80% (24/29) of recipients receiving combined kidney and hematopoietic stem cell transplants from HLA-identical donors [21, 22]. However, in HLA-incompatible settings, this protocol generally results in low-level and transient chimerism, even when higher doses of donor T cells are administered (up to 100 × 10^6^/kg) [22]. Critically, loss of chimerism in this context is often rapidly followed by renal graft rejection [22].

A second strategy, developed at Northwestern University, involves a more intensive conditioning regimen [23], comprising total body irradiation, fludarabine, and cyclophosphamide administered both pre- and post-transplant, along with donor T cells (3.8 × 10^6^/kg). Cyclophosphamide post-transplant, in combination with co-infusion of CD8^+^ TCR^−^ immunomodulatory “facilitator” cells (FCR001), is designed to mitigate the risk of graft-versus-host disease (GVHD) associated with the transfer of mature donor T cells [23]. Over 80% (26/32) of patients in this protocol achieved high and stable levels of chimerism, with successful discontinuation of immunosuppressive therapy in the vast majority (25/26) [24]. In this context, the establishment of full donor chimerism represents the most reliable biomarker of successful tolerance induction [25].

Nevertheless, two major adverse events have emerged as limitations to widespread implementation. Three patients experienced severe infections resulting in graft loss (n = 2) or death (n = 1) [26]. In addition, two cases of GVHD occurred, one of which was fatal, and the other developed into chronic GVHD [26]. These findings emphasize both the necessity and the inherent risk of robust GvH reactivity for maintaining high-level chimerism in the absence of myeloablation [20].

Regarding GVHD risk mitigation, Alice Bertaina and colleagues at Stanford recently reported a novel approach in three patients with Schimke immuno-osseous dysplasia, a syndrome characterized by severe combined immunodeficiency, skeletal dysplasia, and early-onset glomerular kidney failure [27]. This condition is caused by mutations in SMARCAL1, a gene involved in DNA repair, rendering patients highly vulnerable to cytoreductive treatments and increasing mortality following hematopoietic stem cell transplantation [28]. The Stanford protocol reduces this risk through the use of reduced-intensity conditioning and grafts depleted of TCRαβ^+^ T cells and CD19^+^ B cells. All three patients achieved full donor hematopoietic chimerism and maintained excellent renal graft function from the same donor, in the complete and sustained absence of immunosuppressive therapy [27]. The investigators propose expanding this protocol to broader patient populations beyond those with inborn errors of immunity and hematopoiesis [27]. However, favorable outcomes with TCRαβ/CD19-depleted grafts have so far been primarily observed in patients with inborn errors of immunity [29].

Additionally, the case of a child with Schimke syndrome who spontaneously developed acute GVHD and full hematopoietic chimerism following isolated kidney transplantation illustrates the unique pathophysiological context of this condition [12]. This case highlights how host cells in this syndrome, due to their limited proliferative capacity and functional impairment, are at a competitive disadvantage, particularly under immunosuppressive therapy, which favors engraftment of donor-derived hematopoietic stem cells [29, 30].

Collectively, these pilot studies indicate that in patients without preexisting immune deficiency, achieving and maintaining high-level, durable hematopoietic chimerism in the context of HLA incompatibility and without myeloablation requires a degree of GvH reactivity that may carry life-threatening complications.

## Induction of Mixed and Transient Donor Hematopoietic Chimerism

Mixed chimerism refers to the coexistence of donor- and recipient-derived hematopoietic cells in the peripheral blood and indicates the preservation of the recipient’s hematopoietic system [20]. In laboratory mice, stable mixed chimerism can be readily achieved through the administration of donor hematopoietic stem cells in combination with various tolerance-inducing regimens. Historically, the foundation for a clinically translatable strategy was laid using non-myeloablative conditioning that combined cytoreductive agents with thymic irradiation [31]. To mitigate treatment-related toxicity, cytoreduction was successfully replaced in murine models by either co-stimulatory blockade [32] or regulatory T cell-based therapy [33].

However, in humans and non-human primates, prior exposure to pathogens leads to the development of alloreactive memory T cells via heterologous immunity, which impairs the induction of mixed chimerism through immunomodulation alone [6, 34]. The first clinical protocol for tolerance induction via mixed chimerism therefore incorporated siplizumab, an anti-CD2 monoclonal antibody that effectively targets memory T cells [35]. Notably, both siplizumab and alefacept are able to inhibit the expansion of CD2^high^ CD28^−^ pro-inflammatory T cells, which are resistant to CTLA-4-Ig [36, 37]. The development of new agents targeting memory T cells, such as OX40-specific antibodies, may ultimately restore the tolerogenic potential of co-stimulatory blockade in humans, a mechanism currently best demonstrated in murine and non-human primate models [38]. In this context, it is important to highlight the recent communication at the ESOT 2025 congress regarding the first use in humans (NCT07020156) of a monoclonal anti-OX40 antibody (OX118).

Under the leadership of the Massachusetts General Hospital (MGH) team, the mixed chimerism protocol was successfully translated to non-human primates [39] and humans [40, 41]. However, in contrast to murine models, the level and duration of donor chimerism achieved in these species were substantially lower and more transient (lasting only a few weeks). This short-lived chimerism was associated with reduced tolerance efficacy: three of the first ten patients developed *de novo* donor-specific antibodies (DSAs) or acute rejection episodes, precluding immunosuppression withdrawal. Among the remaining seven patients, three had to resume immunosuppression due to chronic rejection or recurrence of native kidney disease [26].

Several modifications have since been introduced to enhance protocol efficacy and compensate for the unavailability of specific therapeutic agents. The inclusion of four doses of rituximab helped prevent *de novo* DSA development, which had been observed in early patients [41]. More recently, the protocol was adapted to address “chimerism transition syndrome,” characterized by acute kidney injury and chimerism loss during rapid immune reconstitution. The revised MGH protocol now includes fludarabine, a reduced dose of cyclophosphamide, and omits post-transplant rituximab [1]. In parallel, the PANORAMA trial (NCT04803006), led by Joshua Weiner at Columbia University, is investigating modified siplizumab dosing to enhance memory T cell depletion, with encouraging preliminary results [1].

At the Samsung Medical Center, where siplizumab is unavailable, the protocol was adapted using anti-thymocyte globulin (ATG) instead. Infectious complications, including BK virus nephritis, prompted dose reductions of both fludarabine and ATG, and a switch from tacrolimus to sirolimus at 1-month post-transplant [42]. In this Korean cohort, immunosuppression was discontinued for over 1 year in five of eight patients. However, one patient experienced T cell-mediated rejection following a respiratory infection, highlighting the fragility of the tolerogenic state [42].

Mechanistic studies have demonstrated a biphasic process in tolerance induction: initial enrichment of regulatory T cells during post-induction lymphopenia [43], followed by progressive deletion of alloreactive T cell clones over time (Figure 1) [44]. The renal allograft likely contributes to this functional inactivation of donor-specific responses (Figure 1). Indeed, patients who received hematopoietic stem cells under the same induction regimen but without a kidney transplant retained anti-donor T cell reactivity upon chimerism loss [45], unlike those with combined kidney-bone marrow transplantation [44]. This hypothesis is supported by observations in non-human primates: combined (simultaneous or sequential) heart and bone marrow transplantation from the same donor failed to induce tolerance [46]. In contrast, triple transplantation of heart, kidney, and bone marrow from the same donor, using identical conditioning, resulted in higher levels of donor chimerism, prevented the formation of DSAs and anti-donor cytotoxic responses, and most importantly, enabled successful withdrawal of immunosuppression [46]. This kidney-specific protective effect was associated with an accumulation of regulatory T cells within the renal graft, suggesting their role in local suppression and potentially in the deletion of alloreactive clones [43, 46, 47].

In line with these findings, during the Sixth International Workshop on Clinical Transplantation Tolerance, several investigators reported the presence of organized infiltrates enriched in FOXP3^+^ regulatory T cells within the grafts of operationally tolerant patients [1]. These structures are reminiscent of regulatory tertiary lymphoid organs observed in renal allografts of tolerant mice [48]. Advances in spatial transcriptomics may soon clarify the prognostic and mechanistic significance of these structures [1].

In conclusion, protocols based on mixed chimerism have yielded mixed results, with variable and often temporary efficacy in inducing tolerance. Further optimization of immunomodulatory regimens accompanying hematopoietic stem cell transplantation will be required to enhance both their clinical effectiveness and safety profile.

## Thymus Transplantation

**FIGURE 2:**
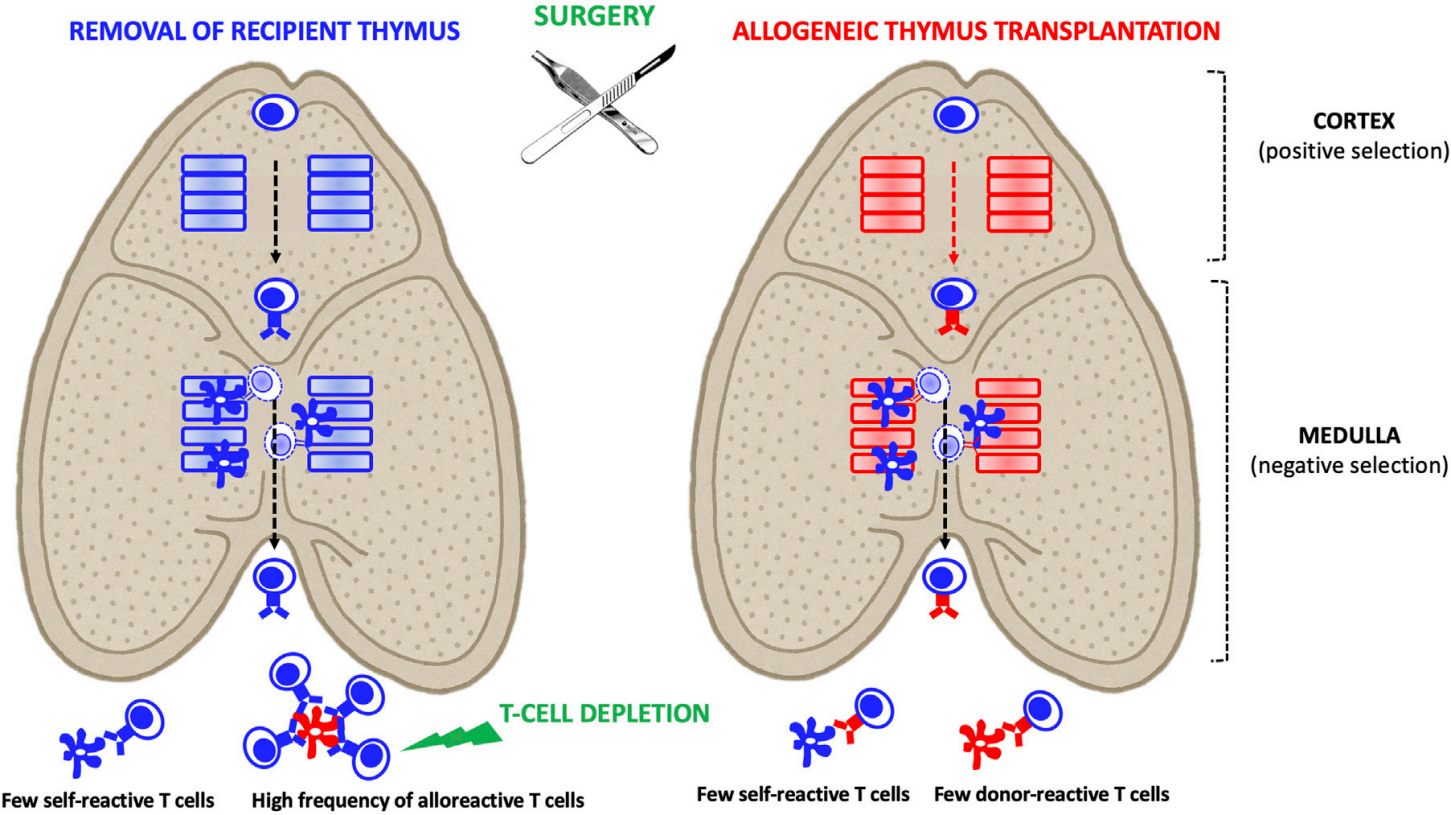
Allogeneic Thymus Transplantation: In the recipient thymus, thymocytes derived from T cell progenitors undergo positive selection by cortical epithelial cells. This positive selection process determines the T cells’ restriction to self-HLA antigens (depicted by a blue TCR) that were recognized in the cortex. Subsequently, developing thymocytes undergo negative selection in the medulla, where those that strongly react to self-antigens presented by medullary epithelial cells and dendritic cells are eliminated. As a result, mature thymocytes exiting the thymus are devoid of self-reactive T cells but may still include alloreactive T cells. In the context of allogeneic thymus transplantation, recipient-derived thymocytes are positively selected on donor HLA molecules (depicted by a red TCR). As they migrate through the medulla, they undergo negative selection, eliminating those that strongly react to recipient HLA (presented by medullary dendritic cells) or donor HLA (presented by medullary epithelial cells). Consequently, the mature thymocytes that exit the donor thymus are tolerant to both recipient and donor antigens. Combining thymus and organ transplantation from the same donor represents a potent strategy to induce immune tolerance. However, two key conditions must be met to achieve this: 1- The recipient’s native thymus must be removed to eliminate a source of donor-reactive T cells. 2- Pre-existing peripheral donor-reactive T cells generated before thymus transplantation must be eliminated.

Thymus transplantation enables the induction of central, donor-specific immune tolerance, particularly when the donor is juvenile, provided two key conditions are met (Figure 2). First, the recipient must undergo thymectomy to ensure that all developing thymocytes are educated within the donor thymic microenvironment [49]. Second, profound peripheral lymphodepletion is required to eliminate pre-existing alloreactive T cells generated prior to thymus transplantation [49, 50].

In murine models, transplantation of a neonatal donor thymus under the kidney capsule of a thymectomized and lymphodepleted recipient enables long-term acceptance of a heart graft from the same donor strain without the need for ongoing immunosuppression [50]. However, the critical role of thymic vascularization in maintaining the tolerogenic function of the thymic epithelium became apparent when this approach was translated to large animal models. To address this, various surgical techniques have been developed to optimize thymic graft perfusion.

One such strategy, known as the “thymokidney” approach, involves transplanting the donor’s thymus under the capsule of one of their own kidneys several weeks prior to allogeneic transplantation [51]. This interval allows the thymus to revascularize and regain functional capacity in an autologous environment before subsequent transplantation of the composite “thymokidney” graft. This approach was notably employed by Robert Montgomery’s team during the first porcine thymokidney xenotransplant into brain-dead human recipients [52]. Thymic perfusion may also be preserved through microsurgical anastomosis of donor thymic vessels [49], or via *en bloc* transplantation of combined thymic and cardiac grafts.

Nevertheless, transplantation of an intact thymus that retains mature donor-derived thymocytes carries a significant risk of GVHD, especially in immunodeficient recipients. To mitigate this risk in athymic infants, researchers at Duke University developed a strategy involving the transplantation of thymic epithelial tissue devoid of donor thymocytes. These thymocytes are eliminated by culturing thymic slices for approximately 10 days prior to implantation. This approach, now FDA-approved under the commercial name RETHYMIC^®^ (allogeneic processed thymus tissue), has dramatically improved outcomes for children with congenital athymia [53]. Its potential to induce alloimmune tolerance has been demonstrated in a rat heart transplantation model [54] and is currently under investigation in clinical transplantation settings [55–57].

## Regulatory Cell Therapy

The iatrogenic risks associated with hematopoietic stem cell transplantation have sparked interest in peripheral immunomodulation strategies using regulatory cell therapies, including T lymphocytes and myeloid cells such as dendritic cells and macrophages [1]. The ONE Study consortium, comprising eight European and American centers, jointly analyzed the clinical and immunological impact of distinct regulatory cell products administered to kidney transplant recipients, using a shared protocol for follow-up [58]. Combined analysis of these trials showed that, when regulatory cell therapy was paired with reduced immunosuppression, infection rates were lower and rejection rates comparable to standard immunosuppressive regimens [58].

A more specific analysis from the German cohort at Charité University Hospital demonstrated the feasibility of generating autologous CD4^+^ regulatory T cell (Treg) products from peripheral blood collected 2 weeks prior to transplantation. Three-year kidney allograft survival reached 100% in both arms of the trial, while 73% of patients who received polyclonal Tregs were maintained on tacrolimus monotherapy [59]. A recent report indicates no graft loss among the 12 patients in the United Kingdom cohort who received polyclonal Tregs, even 7 years after transplantation [1]. Surveillance biopsies from this cohort revealed focal infiltrates enriched in B cell and regulatory gene signatures [60].

These encouraging outcomes have led to the launch of the randomized controlled TWO Study, aiming to enroll 60 patients in two arms. Initially, regulatory cell therapy was scheduled to be administered 6 months after induction with alemtuzumab [61]. Seven patients were treated under this protocol before it was suspended due to concerns about COVID-19-related risks associated with prolonged lymphodepletion [61]. The trial has since resumed with a revised protocol: one arm receives standard immunosuppression after basiliximab induction, while the other includes regulatory cell therapy on day 5 post-transplantation, followed by progressive immunosuppression minimization [62].

In liver transplantation, two clinical trials evaluating donor-specific regulatory cell therapy have yielded contrasting results [63, 64]. A Japanese study achieved complete withdrawal of immunosuppression by 18 months post-transplantation in 70% of patients, with follow-up ranging from 5.4 to 10.4 years [1, 63]. The subsequent multicenter trial (NCT04950842) showed preliminary evidence of FOXP3^+^-enriched lymphoid infiltrates in protocol biopsies, similar to those observed in renal transplantation trials [1]. By contrast, in an American study, 4 out of 5 patients experienced acute rejection during immunosuppression tapering [64]. Notably, timing differed between the two studies: regulatory T cell therapy was administered on day 13 post-transplantation in the Japanese study, whereas in the American trial, it occurred between 2 and 7 years post-transplantation. More importantly, the Japanese protocol included a bolus of cyclophosphamide 1 week prior to Treg infusion, aimed at depleting alloreactive effector T cells that were activated and proliferating immediately following transplantation. This “debulking” effect, combined with regulatory cell therapy, shifts the immune balance in favor of tolerance.

In the American trial, deuterium-labeled cell tracking revealed rapid contraction and disappearance of infused Tregs, likely due to abrupt interleukin-2 (IL-2) withdrawal following an IL-2-rich *ex vivo* expansion phase [64]. Indeed, studies in type 1 diabetes have demonstrated enhanced Treg persistence when low-dose IL-2 is co-administered with cell therapy [65]. However, this strategy is more challenging in transplantation, where IL-2 may simultaneously stimulate effector responses. One liver transplant trial showed significant Treg expansion but also activation of CD8^+^ T cells and NK cells, resulting in unexpectedly high rejection rates and premature trial termination [66]. To enhance IL-2 selectivity for Tregs, multiple pharmaceutical efforts are underway to develop IL-2 muteins with increased affinity for the high-affinity IL-2 receptor (CD25), while minimizing interaction with lower-affinity receptors [67, 68]. Combining regulatory cell therapy with such IL-2 muteins may further amplify therapeutic efficacy.

**FIGURE 3:**
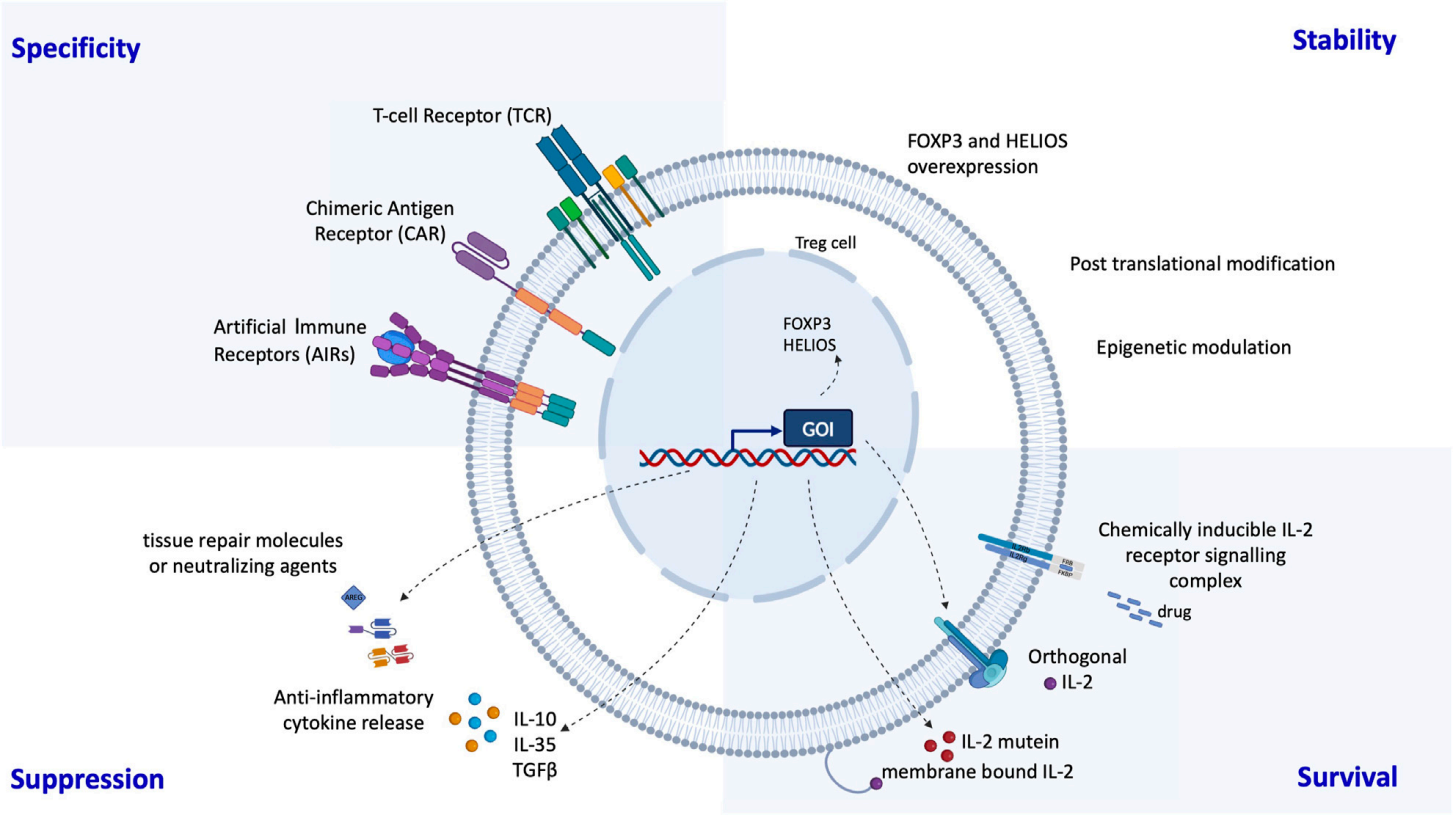
Possibilities to enhance Treg therapies through genetic engineering: Several groups have developed genetic engineering strategies to improve the specificity, stability, survival and suppression of regulatory T cells. Specificity has been greatly enhanced by the addition of chimeric antigen receptors (CARs) or synthetic T cell receptors (TCRs). Stability can be achieved by overexpression of the transcription factors FOXP3 and HELIOS and suppression can be supported by expression of anti-inflammatory cytokines and molecules. The *in vivo* survival of regulatory T cells can be prolonged by making them self-sufficient in a Treg specific IL-2.

Finally, the development of genetically engineered regulatory cells offers highly promising new avenues (Figure 3). Several groups have demonstrated that chimeric antigen receptor (CAR)-redirected Tregs targeting HLA-A2 can suppress alloreactive responses in preclinical transplant models [69, 70]. CAR-Tregs display enhanced suppressive capacity compared to donor-specific Tregs generated via co-culture with donor cells [71]. The ongoing STEADFAST (NCT04817774) and LIBERATE (NCT05234190) trials are evaluating anti-HLA-A2 CAR-Tregs in renal and liver transplantation, respectively [1]. Preliminary findings suggest the presence of FOXP3^+^ regulatory lymphoid structures within renal grafts from CAR-Treg-treated patients [1]. Additional genetic modifications have been proposed to further improve Treg efficacy and resilience. These include conferring resistance to immunosuppressants (e.g., tacrolimus, everolimus, sirolimus) by inactivating FKBP12 [1, 72], and autonomous IL-2 signaling [73]. We are also developing a strategy that harnesses the tolerogenic properties of anti-CD3 monoclonal antibodies [74], in combination with shielded CAR-Tregs that are protected from anti-CD3-mediated clearance via CRISPR-Cas9-based editing (*Blein et al. personal communication*). Additionally, the use of monoclonal antibodies currently under development - such as those targeting CD28 (FR104; NCT04837092) [75] or CD45RC [76] - in combination with targeted regulatory cell therapy, may act synergistically to shift the balance toward immune tolerance. Finally, the transgenic expression of transcription factors involved in the Treg program may help stabilize the Treg epigenetic landscape and reinforce lineage stability in inflammatory environments [77].

## Organ Engineering to Evade the Immune System

An alternative to inducing immune tolerance through immunological reprogramming is the engineering of the graft itself to evade immune detection. Pregnancy provides a compelling demonstration that multiple immunomodulatory mechanisms at the placental interface can create an immunologically privileged zone that remains invisible to alloimmune responses [78]. Failure of these mechanisms can result in placental inflammation resembling transplant rejection [79, 80].

The placenta offers multiple avenues for modulating the immunogenicity of allogeneic transplants, including the epigenetic silencing of polymorphic HLA genes [81] and Th1-skewed chemokine genes CXCL9, CXCL10, and CXCL11 [82]. Additionally, the expression of FasL [83], the enzyme indoleamine 2,3-dioxygenase [84], and immune checkpoint molecules [85] can suppress T cell responses, while specific sialylation motifs on trophoblast proteins inhibit B cell activation [86].

This conceptual framework has already inspired a successful strategy in islet transplantation models in humanized mice [87], non-human primates [88], and more recently, in a first-in-human case [89]. In these studies, three genetic modifications were introduced into allogeneic pancreatic islet cells using CRISPR-Cas12-based gene editing and lentiviral transduction. These modifications involved silencing HLA class I and II molecule expression and overexpressing CD47 [89]. The absence of polymorphic HLA prevents activation of the adaptive immune response (T and B cells), while CD47 expression neutralizes the innate immune response (NK cells and myeloid cells).

The development of normothermic perfusion machines [90, 91], along with rapid advances in cell-specific targeting of viral (lentivirus, AAV) and non-viral vectors [92, 93], opens new avenues for applying these strategies to more complex, vascularized organs beyond pancreatic islets. Even before attempting to render complex organs such as the kidney immunologically privileged, it may be feasible to first engineer the graft endothelium to resist preformed donor-specific antibodies (DSA), thereby mimicking the phenomenon of accommodation [94, 95].

## Current Clinical Implications

To date, no tolerance induction strategy in clinical transplantation has been approved by the FDA or the EMA, nor validated in a completed phase III trial in comparison with standard-of-care immunosuppressive therapies. In other words, protocols designed to induce transplant tolerance have not yet entered routine clinical practice and remain within the realm of research. This observation raises important questions regarding the current limitations and obstacles faced by the strategies developed thus far (Table 1). It also underscores the need for continued research to improve both efficacy and safety (Table 1).

**TABLE 1:** Pros and cons, and advancements of the different tolerogenic strategies.

Tolerance induction strategy	Main Mechanism	Advantages	Limitations	Currently explored new avenues	Challenges before regulatory approval
*Full chimerism*	Robust central tolerance via complete donor-derived hematopoiesis	− Enables successful withdrawal of IS drugs without rejection or development of DSA − Prevents post-transplant recurrence of immune-mediated nephropathies	− Conditioning-related toxicity − Risk of life-threatening GVHD − Delayed immune reconstitution − Increased risk of infections	– Approaches to reduce conditioning intensity and GVHR dependence • Use of Tregs (NCT03943238) • T- and B-cell-depleted HSC graft (NCT05508009) Delayed tolerance approaches, for patients who have already a kidney transplant • NCT03591302 • NCT01649388: terminated by sponsor	– Phase 3 CT required to demonstrate a superior benefit/risk ratio compared to standard IS therapy – • NCT03363945 (HLA-matched LD) • NCT03995901 terminated due to high GVHD rates in initial participants
*Mixed chimerism*	Peripheral tolerogenic mechanisms, via transient, incomplete donor-derived hematopoiesis	− Lower conditioning toxicity − No risk of GVHD − IS drugs withdrawal achieved in some patients	− Less robust tolerogenic effect (rejection or *de novo* DSA may occur upon IS tapering) − Potential recurrence of immune-mediated nephropathies − Risk of chimerism transition syndrome #	Strategies to improve efficacy and safety profile • ECP-DL infusion (NCT07083830) • Combined Treg therapy (NCT03867617) Approaches to mitigate chimerism transition syndrome • Use of fludarabine and avoidance of post-Tx rituximab (NCT04540380) • Enhanced T cell depletion (NCT04803006)	No ongoing Phase 3 CT
*Thymus Tx*	Central tolerance through intrathymic deletion of donor-reactive T cells	− No need of cytoreductive conditioning − Donor thymus can support immune reconstitution after recipient thymectomy	− Requires thymectomy (open-chest surgery) − Profound T cell depletion needed to eliminate preexisting donor-reactive T cells − GVHD may occur following the transplantation of an intact thymus into an immunocompromised recipient − May not promote tolerance to peripheral tissue-specific antigens (as seen in xenotransplantation models)	Combined thymus-kidney transplantation from neonatal donors in KTx recipients (NCT06715865) ##	While CTTI is approved for congenital athymia, there is currently no clinical evidence supporting its tolerogenic efficacy in solid organ transplantation
*Regulatory T cell therapy*	Peripheral tolerogenic effects by shifting the immune balance toward regulation	− No need of cytoreductive conditioning − No risk of GVHD − Potential to enhance Treg function through genetic engineering − May at least allow reduction of IS drug burden	− Risk of lineage instability in inflammatory environments (possible drift toward pro-inflammatory phenotypes) − Short-term persistence after administration − Unknown homing capacity to the transplanted graft − Challenge of generating sufficient cells from patients with end-stage organ failure	Approaches to improve the efficacy and robustness • Combination with donor-derived bone-marrow cells (NCT03867617) • Profound T cell depletion prior to therapy (TWO Study ISRCTN 11038572) • Use of cyclophosphamide before Treg infusion (NCT03577431; NCT03654040) Alternative regulatory T cell types • CD8^+^ Treg cells (NCT06777719) CAR-engineered Tregs • In kidney transplant recipients (NCT04817774) • In liver transplant recipients (NCT05234190)	No ongoing Phase 3 CT currently The RETIRE study is a Phase 2 RCT, comparing Treg therapy combined with reduced IS versus SOC (NCT06552169) Overcoming high manufacturing costs and standardization issues

#: Chimerism transition syndrome: characterized by acute kidney injury, fever, loss of chimerism during reconstitution of the recipient’s immune system.

##: Available information does not clarify whether kidney transplant recipients in the study undergo thymectomy as part of the tolerogenic protocol.

Abbreviations: CTTI, cultured thymus tissue implantation; CT, clinical trial; DSA, donor-specific antibodies; ECP-DL, extracorporeal photopheresis donor lymphocytes; GVHR, graft-vs-host reactivity; GVHD, graft-vs-host disease; IS, immunosuppressive; LD, living donor; SOC, standard of care; Treg, regulatory T cell; Tx, transplantation.

Mixed and transient chimerism protocols have shown variable success, often requiring resumed immunosuppression due to rejection or donor-specific antibodies [96]. Targeting memory T cells combined with immunomodulation may improve outcomes. Full durable chimerism offers more robust tolerance [24] but carries serious risks like GVHD and infections, limiting development [26]. Reducing conditioning intensity and GVH reactivity dependence is a key challenge. Thymus transplantation shows promise in heart and lung transplants, where thymectomy is feasible, but evidence remains limited and its use in abdominal transplants raises safety concerns due to invasive surgery. Moreover, the scientific publication of the first proof-of-concept case remains pending [57]. Polyclonal or donor-specific Treg therapies alone have not safely enabled immunosuppression withdrawal. This emphasizes the need to enhance Treg function (e.g., genetic engineering) while controlling effector immune responses to improve therapeutic success.

## Conclusion

Hematopoietic chimerism induction protocols have demonstrated the possibility of achieving tolerance in clinical transplantation, although sometimes at the cost of excessive iatrogenic risk. The implantation of a juvenile donor thymic epithelial template in a heart transplant recipient, thymectomized during the transplantation procedure, could represent an alternative strategy for central tolerance that should be rigorously evaluated. Finally, “augmented” regulatory cell therapies, through genetic modifications, combined with a targeted strategy of effector cell depletion and immunotherapy favoring the regulatory arm of the immune response, represent very promising strategies.
